# Supplementary material for: The α-Ketoglutarate Dehydrogenase Complex as a Hub of Plasticity in Neurodegeneration and Regeneration
Source: Int J Mol Sci. 2022 Oct 17;23(20):12403. doi: 10.3390/ijms232012403 (PMC9603878; doi:10.3390/ijms232012403)
Supplement: Supplementary file 1 [file ijms-23-12403-s001.zip › ijms-1910749-supplementary.pdf]

Supplementary Table S1. Overview of the subunits in the KGDHC complex.				
**Topic**	**E1k**	**E2k**	**E3**	**E1k- L**
**Name**	α-ketoglutarate dehydrogenase	Dihydrolipoamide succinyltransferase	Dihydrolipoamide dehydrogenase	Oxoglutarate dehydrogenase L
**EC Number**	1.2.4.2	2.3.1.61	1.8.1.4	
**Number of subunits in KGDHC E-Coli**	12 [9]	24 [9}	12 [9]	
**Gene**	OGDH gene on chromosome 7p13-p14 [28]	DLST gene on chromosome 14q24.3 [28	DLD gene on chromosome 7q31.1–7q32 [28]	OGDHL on chromosome 10q11.23 [8]
**Size**	926 amino acids with 40 amino acid leader sequence	453 amino acids	464 amino acids	
**Cofactor**	Thiamine diphosphate	Lipoic acid, Coenzyme A	$FADH_2$ and NAD^+	Thiamine diphosphate
**Metabolic Regulators**	Activated by calcium and ADP and inhibited by ROS, NADH, and ATP [26,27]	Inhibited by Succinyl-CoA, ATP [26,27]	Inhibited by NADH and ATP [26,27]	Activated by calcium and ADP and inhibited by ROS, NADH, and ATP [8]

8. Bunik, V. I.; Degtyarev, D., Structure–function relationships in the 2-oxo acid dehydrogenase family: Substrate-specific signatures and functional predictions for the 2-oxoglutarate dehydrogenase-like proteins. Proteins: Structure, Function, and Bioinformatics 2008, 71, (2), 874-890.
9. Pettit, F. H.; Hamilton, L.; Munk, P.; Namihira, G.; Eley, M. H.; Willms, C. R.; Reed, L. J., α-Keto Acid Dehydrogenase Complexes: XIX. Subunit structure of the Escherichia Coli α-ketoglutarate dehydrogenase complex. Journal of Biological Chemistry 1973, 248, (15), 5282-5290.
26 Bunik, V. I.; Fernie, A. R., Metabolic control exerted by the 2-oxoglutarate dehydrogenase reaction: a cross-kingdom comparison of the crossroad between energy production and nitrogen assimilation. Biochemical Journal 2009, 422, (3), 405-421.
27 Bunik, V. I.; Strumilo, S., Regulation of catalysis within cellular network: metabolic and signaling implications of the 2-oxoglutarate oxidative decarboxylation. Current Chemical Biology 2009, 3, (3), 279-290.
28 Sheu, K. R.; Blass, J. P., The α-Ketoglutarate Dehydrogenase Complex. Annals of the New York Academy of Sciences 1999, 893, (1), 61-78.
